# Evaluation of Serum Vitamin D and C Concentrations in Cats With Gingivostomatitis

**DOI:** 10.1002/vms3.70279

**Published:** 2025-04-02

**Authors:** Farzin Abbasi, Reza Azargoun, Siamak Asri‐Rezaei

**Affiliations:** ^1^ Doctor of Veterinary Medicine Graduate Faculty of Veterinary Medicine Urmia University Urmia Iran; ^2^ Department of Internal Medicine and Clinical Pathology, Faculty of Veterinary Medicine Urmia University Urmia Iran

**Keywords:** ascorbic acid | calcidiol | cat | caudal stomatitis | feline gingivitis | gingivostomatitis

## Abstract

**Aim of Study:**

To investigate serum vitamin C and D concentrations of cats suffering from gingivostomatitis.

**Materials and Methods:**

Serum concentrations of vitamins C and D were evaluated in 30 cats of various breeds, including 15 healthy cats and 15 with gingivostomatitis.

**Results:**

Serum vitamin D concentration in the healthy cats (35.36 ± 15.39 ng/mL) was significantly higher than that of the cats with gingivostomatitis (17.54 ± 10.50 ng/mL) (*p* < 0.05). There was no difference in serum vitamin C concentration between the healthy cats (3.7 ± 0.74 mg/100 mL) and those with gingivostomatitis (3.1 ± 0.72 mg/100 mL) (*p* < 0.05).

**Conclusion:**

The results of this study suggest that cats with gingivostomatitis are predisposed to vitamin D deficiency. Further studies are required to confirm this finding in a larger study sample and to investigate the potential utility of vitamin D supplementation.

## Graphical abstract

This study investigated the levels of vitamins C and D in the serum of cats suffering from gingivostomatitis. Levels of vitamin D were significantly lower in the patient cats when compared to the healthy cats. No significant difference was observed in the levels of vitamin C between the two groups.

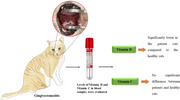

## 1 Introduction

Gingivostomatitis is a clinical term that indicates inflammation and proliferation of the gingiva and oral mucosa (Niemiec 2011). Clinical signs usually include halitosis, anorexia, ptyalism, dysphagia, lack of grooming, difficulty eating and weight loss (Williams and Aller 1992; Johnston 2012). Gingivostomatitis is most commonly detected at three different life stages. First, the immune response associated with primary vaccine courses or the eruption of primary teeth can result in gingivitis in young kittens in the absence of dental plaque. Second, the growth and eruption of permanent teeth can trigger gingival inflammation. Lastly, and most commonly, feline chronic gingivostomatitis (FCGS) is found in older cats with an average age of 7 years (Johnston 2012), the exact pathophysiology of which remains unknown (Southerden 2010). Various aetiologies, such as viral infections, dental plaque bacteria, stress and nutritional and vitamin deficiencies, have been postulated (Lommer 2013; Lee et al. 2020; Peralta and Carney 2019; Dolieslager et al. 2011; Southerden 2010). Currently, there are no definitive treatments available for this condition (Dolieslager et al. 2011).

Vitamin D is a fat‐soluble vitamin that plays many roles in the body. The identification of vitamin D receptors in numerous organs and cells (e.g., macrophages, monocytes, dendritic cells, placental cells, smooth muscle cells, prostate and parathyroid glands, osteoblasts and epithelial cells of the gingiva) has unveiled its involvement in regulating calcium–phosphate metabolism, immune functions, exerting antimicrobial and anti‐inflammatory effects, inhibiting cell proliferation and promoting differentiation. There is much interest in the non‐skeletal effects of vitamin D and the formulation of hypotheses regarding its potential impact on reducing infectious and autoimmune diseases (Pludowski et al. 2013; Mcmahon et al. 2011; Krawiec and Dominiak 2018; Diachkova et al. 2021).

Vitamin C is a water‐soluble enzymatic cofactor containing ascorbic acid and dehydroascorbic acid (Levine et al. 2011). Vitamin C is a crucial nutrient known for its reducing and antioxidant properties, which help neutralize free radicals and serve as a cofactor for enzymes within cells (Carr and Frei 1999; Padayatty et al. 2003). By eliminating excessive reactive oxygen species (ROS), vitamin C is regarded as a significant dietary antioxidant beneficial for periodontal health (Chapple and Matthews 2007). Additionally, this vitamin plays a significant role in inducing the differentiation of periodontal ligament precursor cells, contributing to the prevention and slowing of periodontal disease progression (Yan et al. 2013).

Previous studies have demonstrated the beneficial effects of vitamins on oral health. To date, there has been no reliable evidence showing that vitamins D and C impact gingivostomatitis in cats. Therefore, the present study aimed to examine the serum levels of these vitamins in feline gingivostomatitis and compare vitamin D and C concentrations between mild and severe gingivostomatitis cats and healthy controls.

## 2 Materials and Methods

### 2.1 Animals and Groups

Thirty cats referred to the Veterinary Teaching Hospital of the Urmia University were recruited over a 6‐month period from March 2022 to September 2022. Fifteen cats were referred with gingivostomatitis, and 15 were healthy cats presenting for routine vaccination. The cats have been chosen randomly, and clinical examination, history and laboratory findings confirmed the absence of disease in healthy cats and verified that the patient cats were not affected by other concurrent conditions.

**FIGURE 1:**
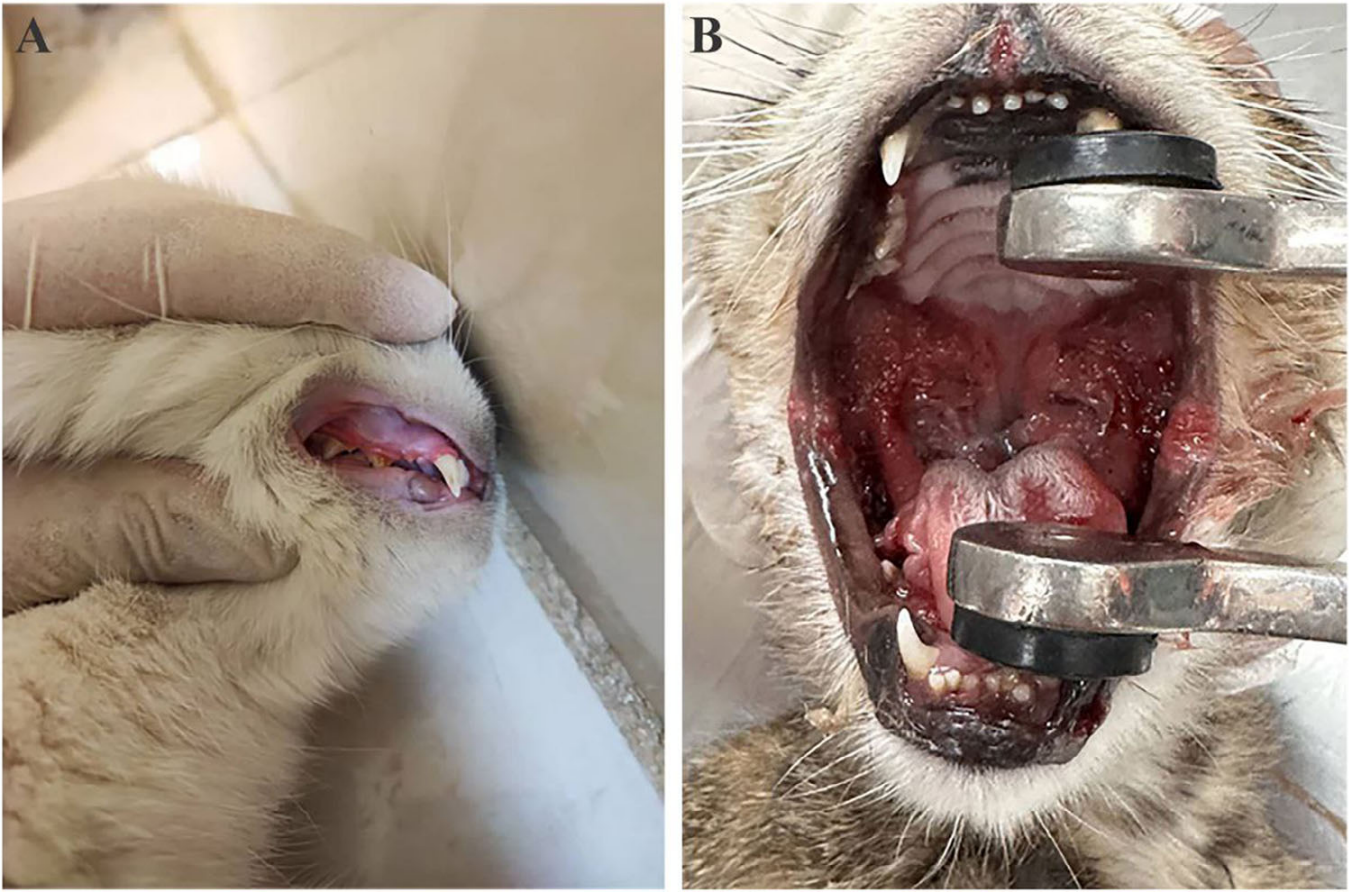
(A) Mild involvement with gingivostomatitis; (B) severe gingivostomatitis.

**Patient group (cases)**: Fifteen cats diagnosed with mild/marginal (*n* = 9) or severe (*n* = 6) gingivostomatitis (Figure 1).

**Control group**: Fifteen healthy cats with no history of gingivostomatitis presenting for routine vaccination.

### 2.2 Sample Collection

Blood samples were collected from each cat by a trained veterinarian using standard venipuncture techniques. Approximately 1–2 mL of blood were drawn from the cephalic vein of each cat using 22 ga sterile needles and syringes and transferred to a clot activator tube. Serum was separated within approximately 20 min of collection following 1500 rpm centrifugation for 10 min and was stored immediately at −80°C until further analysis.

### 2.3 Evaluation of Vitamin D

For the measurement of vitamin D concentration, an enzyme‐linked immunosorbent assay (ELISA) kit manufactured by Monobind Company (kit number 6724‐96) was used according to the manufacturers’ recommended protocols. The optical absorption of standards and samples was then read using an ELISA reader with a 450 nm filter, and the concentration of vitamin D was calculated using the manufacturers recommended protocol, with results expressed in ng/mL.

### 2.4 Evaluation of Vitamin C

Serum vitamin C concentration was measured by the colourimetry method using a spectrophotometer at a wavelength of 700 nm (Kyaw 1978).

Calculation:

$$\begin{array}{ccc} & & \frac{A\left(\mathrm{test}\right)}{A\left(\mathrm{standard}\right)}\times \mathrm{concentration}\;\mathrm{of}\;\mathrm{standard}\left(\frac{\mathrm{mg}}{100\mathrm{mL}}\right)\\ & & \;=\;\mathrm{mg}\;\mathrm{Ascorbic}\;\mathrm{Acid}/100\mathrm{mL} \end{array}$$

### 2.5 Data Analysis

The data were analysed using SPSS software version 22 for Windows, developed by SPSS Inc. in Chicago, IL, USA. The data distribution was assessed for normality using the Shapiro–Wilk test. The independent *t*‐test was used to compare groups, and the data were presented as mean ± standard deviation. Significance was set at *p* value <0.05.

## 3 Results

The frequency of age, breed, gender and body condition score of the studied animals is presented in Table 1.

**TABLE 1:** Patient and control history.

		Age			Breed							Gender				BCS				
Case history												Male		Female						
Study groups		1–6	7–10	Over 10	DSH^a^	BS^b^	P^c^	Si^d^	Hi^e^	SF^f^	TV^g^	Neutered	Intact	Neutered	Intact	1	2	3	4	5
Control (*n* = 15) (50%)		23.33	16.66	10	6.66	6.66	13.33	0	13.33	3.33	6.66	13.33	16.66	3.33	16.66	0	6.66	33.33	3.33	6.66
Patient (*n* = 15) (50%)	Mild (*n* = 9) (30%)	20	3.33	6.66	10	0	10	3.33	6.66	0	0	13.33	6.66	6.66	3.33	0	13.33	13.33	3.33	0
	Severe (*n* = 6) (20%)	10	10	0	16.66	0	3.33	0	0	0	0	3.33	10	3.33	3.33	0	10	6.66	3.33	0

*Note*: The numbers are presented as percentage.

^a^ Domestic short haired.

^b^ British short haired.

^c^ Persian.

^d^ Siamese.

^e^ Himalayan.

^f^ Scottish fold.

^g^ Turkish van.

Univariable statistical analysis revealed a significant difference in serum vitamin D concentration between healthy cats (35.37 ± 15.39 ng/mL) and cats suffering from gingivostomatitis (17.55 ± 10.50 ng/mL) (*p* < 0.05) (Table 2). No significant difference was found in the serum levels of vitamin C between healthy cats (3.7 ± 0.74mg/100 mL) and diseased cats (3.15 ± 0.72 mg/100 mL) (*p* < 0.05) (Table 2).

**TABLE 2:** Mean ± standard deviation of vitamin D and C serum levels in healthy cats and those with gingivostomatitis.

Groups		Vitamin D	Vitamin C
Cats with gingivostomatitis (*n* = 15)	Mild (*n *= 9)	19.68 ± 12.98^b^	3.36 ± 0.63^a^
	Severe (*n* = 6)	15.10 ± 6.88^b^	2.90 ± 0.79^a^
Healthy cats (*n* = 15)		35.36 ± 15.39^a^	3.70 ± 0.74^a^

*Note*: Different letters in each column indicate statistically significant differences (*p* < 0.05).

No significant difference was observed in either vitamin C or vitamin D concentration between cats with mild and severe gingivostomatitis (Table 2).

A complete blood count (CBC) was performed in 12 of 15 cats with gingivostomatitis, variably revealing **leukocytosis**, **neutrophilia** and **anaemia** (Table 3).

**TABLE 3:** Mean ± standard deviation of complete blood count (CBC) parameters in cats with gingivostomatitis (12 out of 15 cats).

Parameter	Results	Reference range
White blood cells (10^9^/L)	19.73 ± 8.64^a^	7.73–18.6
Neutrophils (10^9^/L)	15.33 ± 5.14^a^	3.1–12.5
Lymphocytes (10^9^/L)	3.19 ± 0.86	1.3–7.5
Monocytes (10^9^/L)	0.71 ± 0.11	0.1–1.1
Eosinophils (10^9^/L)	0.45 ± 0.55	0.1–2.2
Red blood cells (10^12^/L)	5.75 ± 2.81^a^	5.9–11.2
Haemoglobin (g/dL)	8.25 ± 3.40	8.2–15.3
Haematocrit (%)	36.55 ± 6.29	31.7–48
Mean corpuscular volume (fL)	39.31 ± 3.09	37–55
Mean corpuscular haemoglobin (Pg)	14.61 ± 2.21	11.8–17.3
Mean corpuscular haemoglobin concentration (g/dL)	28.54 ± 1.30	26.2–35.9
Platelets (10^9^/L)	331.90 ± 76.72	42–630

^a^ Indicates that the value is out of the reference range.

## 4 Discussion

Gingivostomatitis is one of the common problems in cats and can lead to difficulty in eating and oral functions, pain, ptyalism and halitosis. The exact pathophysiology of this problem in cats is still unknown, but possible causes may include viral infections, dental plaques, stress and nutritional deficiencies such as calcium and vitamin deficiencies. There is still no definitive treatment for this condition, and available treatments, despite being costly, too aggressive and time‐consuming, do not yield satisfactory results. Gingivitis sometimes can progress to periodontitis. Periodontitis refers to inflammation in the tissues that support the tooth. This problem can damage the alveolar bone, cementum and periodontal ligament, and it can lead to attachment loss. Unlike gingivitis, periodontitis is irreversible but can often be managed effectively (Wallis and Holcombe 2020).

This study investigates the correlation between vitamin D and C serum levels and gingivostomatitis in cats. However, similar studies have been conducted on the correlation between vitamin D and C levels and periodontitis in people, which are described below.

The results of a study conducted by Bayirli et al. (2020) suggest that low levels of vitamin D can disrupt the response of antimicrobial peptides in periodontal tissues to bacterial challenges. Low levels of vitamin D in the serum can alter the antibacterial activities of periodontal tissues, leading to an inadequate immune response and the advancement of the disease. The mentioned study indicated a correlation between low serum vitamin D levels and reduced expression of antimicrobial peptides in the gingiva of patients suffering from gingivitis (Bayirli et al. 2020).

Gram‐negative bacteria in dental plaques produce lipopolysaccharide endotoxins that stimulate pro‐inflammatory cytokines production (TNF‐α, IL‐1, IL‐6, PGs, matrix metalloproteins). The active form of vitamin D, known as 1,25 (OH)2D3, has anti‐inflammatory and regulatory properties that help reduce pro‐inflammatory cytokines in periodontitis. Additionally, the gingival epithelium has specific vitamin D receptors that improve the function of the epithelial barrier and enhance the innate immune barrier against microbial invasion (Khan and Ahad 2021). Unlike humans and many other species, cats and dogs cannot synthesize vitamin D3 in their skin. Therefore, these two species require dietary supplements of vitamin D. This vitamin has been discovered to have numerous effects all over the body due to the wide range of cells that contain its receptor. When this receptor is activated in humans, it leads to various actions such as the differentiation of immune cells, decreased inflammation and proteinuria, increased insulin secretion and enhanced haematopoiesis (Valdivielso et al. 2009). Martelli et al. (2011) conducted a study to examine the correlation between the polymorphism of vitamin D receptors and periodontal disease in patients suffering from periodontitis. They reported that the polymorphism of vitamin D receptors is linked with the progression of aggressive and chronic periodontitis (Martelli et al. 2011). Boggess et al. (2011) investigated the correlation between the levels of vitamin D and periodontal disease in pregnant women; the results indicated that there is an association between deficiency in serum vitamin D during pregnancy and periodontal disease. Perayil et al. (2015) found that supplements of calcium and vitamin D can improve periodontal health and serve as a non‐surgical option for treating periodontal conditions.

Abreu et al. (2016) conducted a study on the levels of vitamin D in adults with periodontitis in Puerto Rico. Their results revealed that patients had significantly lower vitamin D levels compared to healthy individuals (Abreu et al. 2016). In a study conducted by Antonoglou et al. (2015) on the association between the deficiency of vitamin D and chronic periodontitis, a significant association was reported between serum levels of vitamin D and chronic periodontitis. Van der Velden et al. (2011) studied dietary approaches for treating periodontal issues and found that a positive relationship exists between vitamin D levels of the serum and periodontal health problems. Anbarcioglu et al. (2019) reported a significant correlation between the deficiency of vitamin D and periodontitis in humans. In a study conducted by Ketharanathan et al. (2019), they investigated the relationship between the condition of alveolar bone and serum vitamin D3 levels in patients with periodontitis and healthy controls. Their findings revealed a notable difference in vitamin D levels between the patient group and the control group (Ketharanathan et al. 2019). In line with previous studies, our results demonstrated that deficiency of vitamin D is significantly associated with gingivostomatitis in cats and vitamin D levels were significantly lower in the diseased group compared to the healthy group.

Ascorbic acid might impact periodontal disease by the following mechanisms: (a) Vitamin C deficiency could affect collagen metabolism in the periodontium, potentially hindering tissue regeneration and repair, although there's no experimental backing for this idea. (b) Insufficient ascorbic acid may disrupt bone formation, contributing to periodontal bone loss, but noticeable bone changes occur late in the deficiency. (c) Inadequate ascorbic acid levels might increase oral mucosa permeability to titrated endotoxin and inulin, potentially as ascorbic acid helps enhance the barrier function of the epithelium against bacterial products. (d) Elevated ascorbic acid levels might enhance leukocyte migratory and chemotactic actions, but excessive vitamin C intake could reduce leukocyte bactericidal activity. (e) It seems that ascorbic acid is effective in maintaining the integrity of periodontal blood vessels and supporting their function against bacterial stimuli and wound healing. (f) A deficiency in vitamin C can disrupt the balance of bacteria in plaque and lead to its pathogenicity, although there's no direct evidence supporting this claim (Pavithra et al. 2017).

Kuzmanova et al. (2012) found a correlation between low serum levels of ascorbic acid and periodontitis. A study by Khademi et al. (2014) examined the serum levels of vitamins C, A and E in individuals with recurrent aphthous stomatitis. Their findings indicated that there was no significant difference in the levels of these vitamins when compared to a healthy control group (Khademi et al. 2014). Haidary et al. (2022) conducted a study on chronic gingivostomatitis treatments in cats. They also used vitamin C alongside other medications as a supportive medicine. In the mentioned study, vitamin C was effective as a supportive medicine alongside other medications (Haidary et al. 2022). Brahmavar et al. (2021), after gingival injection of vitamin C and observing a significant improvement in the inflammation at the injection site, reported that vitamin C is effective as an anti‐inflammatory agent to treat gingivitis. Another relevant study conducted in this field is the research by Pussinen et al. (2003). They focused on the association between periodontitis and low serum levels of vitamin C. Consequently, a significant correlation was found between the concentration of vitamin C and antibodies against *Porphyromonas gingivalis* bacteria, as well as periodontitis (Pussinen et al. 2003). A study by Isola et al. (2019) investigated the impact of periodontitis on vitamin C levels of serum and saliva. The results demonstrated a significant association between vitamin C levels of serum and saliva in the periodontitis patients when compared to the control group (Isola et al. 2019). Amarasena et al. (2005) examined the relationship between vitamin C levels of serum and periodontitis in elderly Japanese living in the community. Their findings revealed an inverse association, where a reduction in vitamin C levels corresponded with an increased prevalence of periodontitis. The data analysis indicated that individuals with lower vitamin C levels had a 4% higher occurrence of periodontitis compared to those with higher vitamin C levels (Amarasena et al. 2005). Iwasaki et al. (2012) conducted research on the connection between ascorbic acid levels of serum and periodontitis, and the analysis of results indicated that ascorbic acid deficiency could be associated with periodontitis among elderly Japanese individuals. In agreement with Khademi et al. (2014) findings and contrary to the results reported by Kuzmanova et al. (2012), Haidary et al. (2022), Brahmavar et al. (2021), Pussinen et al. (2003), Isola et al. (2019), Amarasena et al. (2005) and Iwasaki et al. (2012), our results revealed no significant difference between vitamin C serum levels in cats suffering from gingivostomatitis and healthy cats.

One probable explanation for the variance between the outcomes of the present study and previous studies could be the difference in the study species. Previous studies have focused on humans, and the sole source of vitamin C in humans is dietary intake from vitamin C–containing foods and fruits. In contrast, unlike humans, cats do not require dietary vitamin C intake, and their vitamin C requirement is synthesized in the liver. This difference between humans and cats could significantly justify the divergent results obtained in the current study and previous studies.

In a study conducted by Arzi et al. (2021), significant leukocytosis and neutrophilia were reported in cats affected by FCGS (Arzi et al. 2021). Soltero‐rivera et al. (2023) reported the presence of anaemia in some cats with gingivitis and periodontitis. In the present study, CBC test was performed on 12 out of 15 patient cases, revealing mild leukocytosis, neutrophilia and anaemia in some affected cats.

## 5 Conclusion

In general, the current study provides valuable insights into nutritional factors and potential preventive measures related to gingivostomatitis. Our findings suggest that vitamin D is associated with feline gingivostomatitis, and deficiencies of vitamin D could potentially be regarded as a risk factor in the onset of gingivostomatitis. This study revealed no significant association between serum levels of vitamin C and involvement in gingivostomatitis in cats.

This study is subject to several limitations. The sample size was relatively small, potentially reducing the generalizability of the findings. Other factors influencing serum vitamin levels, such as diet and sunlight exposure, were not controlled in this study. Nevertheless, further research studies are needed to determine the effect of vitamin D and other vitamins on feline gingivostomatitis. Unfortunately, due to the lack of consent from some clients, the CBC was performed on only 12 out of 15 patient cases. Due to the lack of consent from the owners of the studied cats, diagnostic imaging and blood biochemistry tests could not be performed on all cats to confirm the health of the healthy cats or rule out underlying diseases in the sick ones. However, clinical examinations, history reviews, conducted tests and diagnostic imaging of some suspected cases largely confirmed the absence of other underlying diseases in these animals. Additionally, due to financial limitations, investigating the probable multifactorial causes of FCG was not feasible.

## Author Contributions

**Farzin Abbasi**: conceptualization, methodology, investigation, writing – original draft, data curation. **Reza Azargoun**: conceptualization, methodology, formal analysis, validation, investigation, writing – review and editing, project administration, supervision. **Siamak Asri‐Rezaei**: writing – review and editing, supervision, visualization, investigation, resources.

## Ethics Statement

The Animal Ethics Committee of Urmia University approved the experiments, and their guidelines were followed (Code number: IR‐UU‐AEC‐3/68).

## Conflicts of Interest

The authors declare no conflicts of interest.

### Peer Review

The peer review history for this article is available at https://publons.com/publon/10.1002/vms3.70279.

## Data Availability

The data that support the findings of this study are available from the corresponding author upon reasonable request.
